# Physical Fitness Perception and Physical Education Enjoyment in 11- to 12-Year-Old Children

**DOI:** 10.3390/children10010068

**Published:** 2022-12-28

**Authors:** Francisco José Borrego-Balsalobre, Francisco Cavas-García, Arturo Díaz-Suárez, Alfonso Martínez-Moreno

**Affiliations:** Department of Physical Activity and Sport, CEI Campus Mare Nostrum, University of Murcia, 30100 Murcia, Spain

**Keywords:** physical activity, motivation, enjoyment, boredom, physical condition, anthropometric

## Abstract

Childhood motor competence plays a fundamental role in long-term adherence to physical activity. Enjoyment levels and self-perception of physical fitness increase motivation, commitment, and participation in physical activity. The objective of this study was to assess the body mass index (BMI), weight percentile, health status, physical fitness, and enjoyment levels of physical activity. A sample of 138 persons, of whom 67 were males and 71 females, with a mean BMI of 20.04 (2.35) answered the questionnaires Physical Activity Enjoyment Scale and the International Fitness Scale–self-report of physical fitness in young people. Tests were administered to measure functional fitness and anthropometric characteristics. Statistical analysis included calculation of Cronbach’s alpha, ANOVA and Pearson’s bivariate test correlations. The data indicate the reliability of the International Fitness Scale. No significant differences by gender were observed for the Physical Activity Enjoyment Scale. The BMI showed that significant positive correlations with 20 m sprint scores (*p* < 0.001, r = 0.367) and significant negative correlations with 6-minute lap scores (*p* < 0.001, r = −0.360) could be caused by an older physiological age. The overall physical fitness level of the children was medium-high, with most of the children enjoying physical education classes and slightly higher enjoyment values for males 40.88 (5.42) than females 40.31 (6.16).

## 1. Introduction

Digitalization, residing in large urban centres and traveling long distances in motor vehicles daily promote physical inactivity, especially in children. To mitigate health risks associated with inactivity, the World Health Organization established global physical activity (PA) recommendations by population group and age range to promote healthy lifestyle habits [1]. It is recommended that children aged 5 to 17 years accumulate 60 min of moderate- to vigorous-intensity PA per day on average [2]. However, PA levels remain low worldwide [3] and are associated with an increased incidence of obesity and risk of noncommunicable diseases. Increasing PA levels is vital to improving the health of future generations [3,4].

Childhood motor competence plays a key role in long-term PA adherence and is predictive of health-related physical fitness (PF) later in life [5]. Positive relationships between increased PA levels in children and improved cardiometabolic risk factors for health, musculoskeletal health, mental health and well-being, cardiorespiratory fitness and reduced risk of unhealthy weight gain are well established [6]. Increased muscle strength in childhood, youth, and middle age is associated with a decreased risk of prediabetes in middle age [7], highlighting the importance of muscle strength throughout life [8]. The acquisition of motor skills in early childhood may be an important prerequisite for children’s participation in PA [9] and lead to increased PA throughout life [10]. Adolescent PA patterns have also been established as determinants of health and well-being in adulthood [11].

The theory of reasoned action [12,13] proposes that factors such as attitude and subjective norms can influence behavioral intentions, which can lead to actual behaviors. While high levels of PF and motor competence are assumed to result from high levels of PA [14], the relationship is often more complex. As a behavior, PA is determined by more psychosocial influences than the personal attributes of PF and motor competence [15]. PA promotes student motivation for learning and academic performance [16]. A significant, positive correlation exists between sports motivation and athletes’ psychological resilience scores across several dimensions [17]. Self-esteem, emotion, and motivation influence perception during sports performance, as well as performance itself [18]. Motivation energizes human behavior and sets a direction for progress [19], while high levels of self-perception are associated with increased motivation and commitment to PA and increased motivation for participation in physical education (PE) [20]. Perceived competence, rather than actual competence, determines motivation to practice sports and PE in children [21] and adolescents [22]. Only children with high motor competence who are aware of their competence are more active than their low-proficiency counterparts [23]. Real PF and self-perceived PF exhibit strong relationships in all their components, including cardiorespiratory capacity, muscle strength, agility, and flexibility [24,25].

In the study of PA behaviors and intrinsic motivation, enjoyment is a relevant factor [26,27,28] as it is understood as a positive attitudinal response towards the sports experience [29] and a key factor influencing sports commitment [30]. Enjoyment can also be conceptualized as a positive affective state, either cognitive or physiological, involving feelings of pleasure and fun associated with performing or fulfilling PA [31,32]. Enjoyment is repeatedly cited as an important correlate or predictor of participation in PA and included in health promotion models and motivation theories [33,34]. Additionally, negative emotions are important predictors when we study subjects who do not regularly participate in PA [35,36]. Meanwhile, PF is defined as a set of attributes that people have or achieve related to their ability to perform PA [37]. PF is a multidimensional construct, consisting of cardiovascular endurance, muscle strength, flexibility, and motor control [38,39]. In childhood and adolescence, PF is an effective marker of health [40]. Young people (children or adolescents) with low motor competence tend to report lower levels of several PF indices compared to peers with greater motor competence [41,42,43,44].

The need for the study is highlighted by the importance of motor competence as a catalyst in long-term adherence to PA [5] and specifically perceived competence, rather than actual competence, and determines motivation to engage in sport and physical education in children [21] and adolescents [22], with a strong relationship between actual and self-perceived PF [24,25]. Moreover, enjoyment is a relevant factor [26,27,28], as a positive attitudinal response to the sport experience [29] and a key factor influencing sport engagement [30].

We propose several research questions: What is the level of health and PF of preadolescent schoolchildren (11 to 12 years old)? Do they enjoy or get bored in PE classes? Does their perception match their true PF level? Is there a relationship between their PF perception and PE enjoyment? Assessing physical fitness in young people has clinical, educational, and public health relevance [45], especially in this age group, where the practice of PE is beginning to decline and necessitating investigation with various methods [46]. Therefore, the aim of this study is to analyse and assess body mass index (BMI), weight percentile, health, fitness (self-reported versus field tests), and enjoyment of PA. In addition, we set out to explore the relationship between pre-adolescent self-perception of PA and enjoyment of PA.

## 2. Materials and Methods

### 2.1. Participants and Procedure

An a priori power analysis was conducted using the G*Power version 3.1.9.7 to determine the minimum sample size required to test the study hypothesis. Results indicated the required sample size to achieve 80% power for detecting a medium effect, at a significance criterion of α = 0.05, was N = 128 for an ANOVA statistical test. At the same time, results indicated the required sample size to achieve power (1-β) = 0.95, at a significance criterion of α = 0.05 and correlation p H1 = 0.3, was N = 138 for the Correlation bivariate normal model statistical test. 

The convenience sample for the study consisted of 138 sixth grade primary school students from three schools in the region of Murcia, Spain. The participating students were 48.6% (n = 67) male and 51.4 % (n = 71) female, with a mean age of 11.21 years (±0.409). All necessary permits were requested from the management bodies of the educational centres, and families were informed of the protocols and objectives of the study. Parents were required to sign an informed consent form for their children to participate. The tests were conducted on two separate days of the same week, coinciding with PE sessions in the centre’s facilities. Anthropometric data were collected, and questionnaires were completed on the first day, while the physical fitness tests were conducted on the second day. Researchers answered any student questions, respecting the confidentiality and anonymity of the students. The data collection process was approved by the Research Ethics Commission of the University of Murcia (Spain) and was conducted in accordance with the Declaration of Helsinki. 

### 2.2. Measures and Instruments

A range of physical tests were directly provided by the researchers, and two questionnaires were completed by the schoolchildren under the supervision of the researchers. Sociodemographic data were collected via a questionnaire and anthropometric characteristics measured included weight, height, and BMI. Body weight was measured with a digital scale (Tanita TBF-300) and height was measured with a portable stadiometer (Seca 213). Height and weight were used to calculate weight percentiles and BMI, using the formula: BMI = weight (kg)/height (m)^2^. The percentiles previously proposed as thresholds were used to classify children into the following obesity categories: (1) underweight (≤14.5); (2) normal weight value (NVW) (>14.5 years ≤ 20); (3) overweight (>20 years ≤ 23); and (4) obese [>23] [47,48].

A range of tests of physical fitness (TPF) were conducted with the aim of obtaining reliable and objective information on the PF of children aged 5 to 12 years: (TPF 1) standing broad jump; (TPF 2) two leg jump, 7 m; (TPF 3) single foot hop (7 m); (TPF 4) tennis ball throw; (TPF 5) 1 kg ball push; (TPF 6) espalier climb; (TPF 7) 10 × 5 m relay; (TPF 8) 20 m sprint; and (TPF 9) 6 min lap run (reduced Cooper test). Higher test scores indicate better performance, and hence, PF, for all nine tests [49].

The Spanish version [27] of the physical activity enjoyment scale (PACES) [50] was applied. The scale consisted of 16 items preceded by the phrase “when I’m active…” and rated enjoyment from the highest level (e.g.,“likes”, “it’s very exciting,” and “I find it enjoyable”) to the lowest (e.g., “I’m bored”, “I don’t like it”, and “it frustrates me”). The answers were collected on a Likert scale from: (1) strongly disagree; (2) disagree; (3) neither agree nor disagree; (4) agree; and (5) strongly agree. Factor analysis with enjoyment of physical activity (EPA) and boredom with physical activity (BPA) was used to differentiate between enjoyment of and boredom with the exercise [51]. 

The International Fitness Scale (IFIS) was developed in Spain by the HELENA12 group and validated in Spanish [46]. The aim of the questionnaire is to obtain a self-report of PF and is structured in five subdimensions: (IFIS 1) general PF; (IFIS 2) cardiorespiratory capacity; (IFIS 3) muscular strength; (IFIS 4) speed/agility; and (IFIS 5) flexibility. The answers were collected on a Likert scale that evaluated the level of PF from: (1) very bad; (2) bad; (3) acceptable; (4) good; to (5) very good. The sum of all five subdimensions indicates the total value of the self-reported, perceived PF.

### 2.3. Data Analysis

Basic descriptive methods (standard deviation, range, etc.) were used for descriptive statistical analysis of the measured variables. Cronbach’s alpha was applied to assess the reliability of the scales, with values of 0.70 for the IFIS scale and 0.79 for the PACES scale. Because the sample size was over 50, the Kolmogorov–Smirnov test was performed to verify a normal distribution of the data for each variable. A mean analysis of independent samples was performed for the gender variable through a student’s t-test and for the weight percentile category variable through an ANOVA. Bivariate correlations between numerical variables were established through the Pearson test to determine possible interactions between variables. Data analysis was conducted using the Statistical Package for Social Science^®^ software, version 28 (SPSS^®^, Chicago, IL, USA), with a significance level of α = 0.05 and a perfect correlation coefficient when r = |1|, very strong when |0.8| ≤ r < |1|, strong when |0.5| ≤ r < |0.8|, weak when |0.3| ≤ r < 0 and null when r = 0.

## 3. Results

Total IFIS scores were similar across genders (Table 1). The highest IFIS 3 (strength) scores were observed in males, while higher IFIS 5 (flexibility) scores were observed in females. By weight percentile, the NVW group reported maximum self-perceptions (very good) in IFIS 2 (cardiorespiratory resistance), IFIS 5 (flexibility) and Total IFIS. For IFIS 3 (strength) and IFIS 4 (speed/agility), the NVW and overweight groups reported similar values (very good), while the obese group reported higher values for IFIS 3 and lower values for IFIS 4. Males reported the highest PACES scores of 5 (strongly agree) for EPA, while males and females showed similar results when they indicated 1 (disagree). By weight percentile, the overweight and obese groups showed similar results for EPA, with the NVW group obtaining the highest scores. Meanwhile, the NVW and overweight groups had similar BPA scores (strongly disagree) that were higher than the scores of the obese group.

Females had higher average BMI than males, while males scored higher than females for TPFs 1, 3, 4, 6 and 9 (Table 2). Males and females scored similarly for TPFs 2 and 5, while females scored higher than males for TPFs 7 and 8. The underweight and NVW groups scored higher in TPFs 2, 3, 7 and 9 than other weight percentiles. The obese group scored highest for TPFs 4 and 5, while mean scores for TPFs 1, 6 and 8 were similar for the NVW and overweight groups.

### 3.1. Analysis by Gender

No statistically significant differences by sex or weight percentile were observed for IFIS and the two PACES factors (EPA and BPA). Neither were they observed for BMI nor for TPFs 2, 3, 5, 6 and 7. Significant differences by gender were observed for TPF 1 (standing broad jump) scores (*p* = 0.05), assuming equal variances of the Levene test, and for TPF 4 (tennis ball throw) scores (*p* < 0.001), not assuming equal variances. Significant differences by gender were also observed for TPF 8 (20 m sprint) (*p* < 0.001) and TPF 9 (6 min lap run) (*p* = 0.001), again with the assumption of equal variances for the Levene test. Table 3 shows the estimated magnitude of the effect of gender on scores for TPFs 1, 4, 8 and 9. Gender had a medium effect on TPFs 1, 8 and 9 and a large effect on TPF 4. Males had higher scores than females for TPFs 1, 4 and 9, while females had higher scores than males for TPF 8.

### 3.2. Analysis by Weight Percentile

BMI (*p* < 0.001), TPF 5 (*p* = 0.026), TPF 7 (*p* = 0.039) and TPF 9 (*p* = 0.014) varied significantly among weight percentiles, while no significant differences were observed for the remaining TPFs (Table 4). The effect size by weight percentile categories was large for the BMI and medium for all other variables (Table 5). Additionally, obese children had a higher BMI than the other weight percentile categories, with significant relationships between all categories. The underweight group had higher TPF 3 scores than the NVW and overweight groups. The NVW and overweight group had higher TPF 6 scores than the underweight groups, with no significant relationships with the obese category. However, for TPF 7, the groups that were overweight or obese performed worse than the underweight and NVW groups. For TPF 9, there were only significant differences between the NVW group and the obese groups, with the NVW group scoring higher than the obese group.

### 3.3. Analysis of Bivariate Correlations between BMI, PACES (EPA-BPA) and TPF

Table 6 shows the relationships of the quantitative variables through Pearson’s coefficient. The BMI showed a significant, positive relationship with TPF 8, and a significant, negative relationship with TPF 9. EPA showed significant, positive relationships with IFIS, TPF 8 and 9, and a significant, negative relationship with BPA. BPA showed a significant, positive relationship with TPF 1 and a significant, negative relationship with the IFIS. The IFIS was significantly, positively correlated with TPFs 3 and 6. Multiple significant, positive correlations were observed among TPFs: TPF 1 with TPFs 4 and 9; TPF2 with TPFs 3, 6, 7 and 8; TPF 3 with TPFs 6 and 7; TPF 4 with TPF 9; TPF 6 with TPF 7; and TPF 7 with TPF 8. Additionally, multiple significant, negative correlations were observed among TPFs: TPF 1 with TPFs 5 and 8; TPF 2 with TPF 5; TPF 3 with TPF 9; TPF 4 with TPFs 7 and 8; TPF 5 with TPF 7; and TPFs 7 and 8 with TPF 9.

## 4. Discussion

The objective of this study was to analyze and assess BMI, weight percentile, health level, physical fitness (self-reported versus field test) and the enjoyment level of physical activity, as well as the possible relationship between preteens’ perception of their physical fitness and the motivational role of physical education. The IFIS scale, as well as objective measures, have previously established validity for classifying subjects of different age groups according to health-related physical fitness [45,46,52]. In our study, males obtained higher self-reported fitness scores than females, as in the studies by Ortega et al; Sanchez-López et al; Navarro et al; Olivares et al [45,46,53,54]. The level of concordance between self-reported fitness and that obtained by field testing found in our study is consistent with levels previously reported in adolescents and young adults by Ortega et al., 2011–2013; Sánchez-López et al. [45,46,52]. Children often overestimate their abilities because of cognitive limitations that prevent them from differentiating between their ideal in terms of competence and their own reality [55]. There were significant relationships between IFIS and TPFs 3 and 6, coinciding with Muros et al. [56]. Both TPFs 3 and 6 have a high influence of muscle strength, of both leg and arm, and strength is possibly the easiest element for subjects to perceive.

The analysis of the PACES: EPA/BPA revealed an overall score of 55.01, in line with Giuriato et al. [57]. No statistically significant differences by gender were observed, coinciding with the results of Navarro et al.; Barreal-López et al.; Espinoza-Cortez et al; and Iturricastillo and Yanci [53,58,59,60] in high school students. However, males indicated slightly higher levels of EPA compared to females, as indicated by Chamero and Fraile-García [26], corroborating the observation that males tend to have a higher degree of enjoyment than females (Carroll et al. [61]). Other studies found significant differences in PACES: EPA/BPA as a function of gender such as those of Moreno et al.; Yli-Piipari [62,63], even though the ages studied were older than those of the participants in this study. These data could allow for rapid detection of preadolescents at risk of dropping out of physical activity, an age where the level of abandonment is high [64] due to lack of fun, among other factors [65]. Providing varied, motivating practices that promote authentic and effective learning is necessary for encouraging continued participation in physical activity [66].

Obesity strongly influenced the four components of the fitness and ability tests in previous research [67]. In our study, there were statistically significant and positive differences between BMI and TPF 8 (20 m sprint), coinciding with Utari; Sedeaud et al. [68,69], yet contradicting the results of Septadina and Suciati [70]. BMI was also significantly, negatively correlated with TPF 9 (6 min. lap run), coinciding with Septadina and Suciati; Carnethon et al. [70,71]. There were multiple significant, positive correlations among TPFs: TPF 1 (standing broad jump) with TPFs 4 and 9; TPF2 (7 m two leg jump) with TPFs 3, 6, 7 and 8; TPF 3 (single foot hop, 7 m) with TPFs 6 and 7; TPF 4 (tennis ball throw) with TPF 9; TPF 6 (wall bar climb) with TPF 7; and TPF 7 (10 × 5 relay run) with TPF 8. Additionally, TPF 1 was significantly, negatively correlated with TPFs 5 and 8, which differs from the results of Musa et al. [72], possibly because the ages are different from those in that research.

Due to the cross-sectional nature of the study and, therefore, the inability to establish causation, as well as the small sample size from a specific location in Spain, the data cannot be extrapolated. Additionally, maturation processes are not the same in all individuals, which is a confounding factor that needs to be minimized or eliminated in future studies. A practical implication of this research is that interventions in physical education and physical activity should be directed concomitantly towards motor competence and health-related physical fitness to promote lifelong adherence to the practice of physical activity. An institutionalised record of data would be appropriate for monitoring and adapting programmes for pupils as they move through the different stages of education.

## 5. Conclusions

The physical fitness level of the children sampled was good and very good, with most enjoying physical education classes and slightly higher enjoyment values in males than females. Student self-perception of their physical fitness level coincided with the field tests performed. Children in the underweight category had the highest physical fitness scores in the two-leg jump, single foot hop, wall bar climb and 10 × 5 relay run tests, while children in the overweight category scored the highest in the 1 kg ball push. Children in the obese category scored highest in the standing broad jump, tennis ball throw and 20 m sprint tests. These results highlight the need for diverse and motivating approaches in physical education classes for students to acquire commitment to the practice of physical activity and maintain healthy habits throughout life.

## Figures and Tables

**Table 1:** Descriptive statistics of the International Fitness Scale (IFIS) for self-reported fitness in youth and physical activity enjoyment scale (PACES) scores by gender and weight percentile. PACES scores are divided into enjoyment of physical activity (EPA) and boredom with physical activity (BPA).

		Fr	1	2	3	4	5	Min–Max.	Mean (SD)
**IFIS Total**		138	2.65%	5.05%	17.15%	29.25%	45.9%	2–10	4.11 (0.69)
	Male	67	3.0%	6.2%	19.4%	22.7%	48.7%	1–5	4.08 (0.68)
	Female	71	2.3%	3.9%	14.9%	35.8%	43.1%	1–5	4.14 (0.71)
	Underweight	3	0.0%	0.0%	60.0%	26.7%	13.3%	4–4	3.93 (1.16)
	NVW	85	3.1%	4.1%	15.5%	27.2%	50.1%	2–5	4.17 (3.49)
	Overweight	39	2.6%	8.2%	16.4%	31.8%	41.0%	2–5	4.01 (3.74)
	Obese	11	0.0%	3.6%	29.1%	30.9%	36.4%	3–5	4 (2)
**PACES (EPA) Total**		138	0.0%	1.45%	2.15%	12.8%	83.6%	29–80	40,6 (5.79)
	Male	67	0.0%	1.5%	1.5%	6.0%	91.0%	11–45	40.88 (5.42)
	Female	71	0.0%	1.4%	2.8%	19.6%	76.2%	18–45	40.31 (6.16)
	Underweight	3	0.0%	0.0%	0.0%	33.3%	66.7%	39–45	43 (3.46)
	NVW	85	0.0%	1.2%	3.5%	10.7%	84.6%	11–45	41.08 (5.72)
	Overweight	39	0.0%	2.6%	7.7%	15.5%	74.2%	18–45	39.38 (6.51)
	Obese	11	0.0%	0.0%	0.0%	27.3%	72.7%	37–45	40.36 (3.59)
**PACES (BPA) Total**		138	29.8%	26,25%	28.2%	10.9%	4.85%	14–69	14,41 (7.66)
	Male	67	30.0%	30.0%	24.0%	10.5%	5.5%	7–35	14.22 (7.94)
	Female	71	29.6%	22.5%	32.4%	11.3%	4.2%	7–34	14.59 (7.38)
	Underweight	3	100.0%	0.0%	0.0%	0.0%	0.0%	7–7	7 (0)
	NVW	85	29.4%	25.9%	24.8%	13%	6.9%	7–35	14.84 (8.18)
	Overweight	39	28.2%	28.2%	33.3%	7.7%	2.6%	7–34	13.67 (6.81)
	Obese	11	18.2%	27.3%	45.4%	9.1%	0.0%	7–28	15.82 (6.06)

*NOTE: Fr: frequency. Values: 1 (strongly disagree), 2 (disagree), 3 (neither agree nor disagree), 4 (agree), 5 (strongly agree).*

**Table 2:** Descriptive statistics of body mass index (BMI) and tests of physical fitness (TPF) scores by gender and weight percentile.

				TPF								
			BMI	1	2	3	4	5	6	7	8	9
TOTAL (n = 138)		mean	20.04	1.19	3.41	2.99	14.91	3.95	12.99	21.41	4.55	1055.76
		SD	2.35	0.17	0.66	0.75	3.59	0.62	0.75	2.43	0.51	153.63
**Sex**												
Male (n = 67)		mean	19.86	1.23	3.41	3.12	16.30	3.96	13.12	21.07	4.38	1100.75
		SD	2.51	0.19	0.70	0.95	3.78	0.64	0.95	2.50	0.47	172.83
Female (n = 71)		mean	20.23	1.15	3.42	2.88	13.60	3.94	12.88	21.75	4.73	1013.31
		SD	2.20	0.15	0.63	0.48	2.88	0.61	0.48	2.34	0.50	119.45
**Weight percentile**												
Underweight (n = 3)		mean	14.43	1.36	4.35	4.32	13.83	3.23	14.32	24.07	4.14	1105.00
		SD	0.43	0.04	1.64	3.22	4.16	0.25	3.22	3.55	0.03	153.05
NVW (n = 85)		mean	18.95	1.18	3.42	2.95	15.01	3.91	12.95	21.58	4.57	1082.29
		SD	1.09	0.17	0.55	0.71	3.80	0.57	0.71	2.32	0.48	161.75
Overweight (n = 39)		mean	21.61	1.19	3.37	2.93	14.33	4.09	12.93	20.68	4.57	1028.72
		SD	0.88	0.17	0.77	0.42	3.16	0.61	0.42	2.52	0.58	99.45
Obesity (n = 11)		mean	24.55	1.27	3.32	3.26	16.55	3.96	13.26	20.04	4.58	933.18
		SD	3.35	0.22	0.66	0.39	3.17	0.93	0.39	2.13	0.55	187.81

*NOTE: SD: standard deviation. TPFs: (1) standing broad jump; (2) two leg jump (7 m); (3) single foot hop (7 m); (4) tennis ball throw; (5) 1 kg ball push; (6) wall bar climb (7) 10 × 5 relay run; (8) 20 m sprint; (9) 6 min lap run (reduced Cooper test).*

**Table 3:** Magnitude of the effect of gender differences on scores for tests of physical fitness (TPF) 1 (standing broad jump), 4 (tennis ball throw), 8 (20 m sprint), and 9 (6 min. lap run).

	Mean		*p*	1-β	*d*
	Male = 67	Female = 71			
TPF 1	1.23	1.15	0.05 *	0.771	0.463
TPF 4	16.30	13.60	<0.001 **	0.996	0.804
TPF 8	4.38	4.73	<0.001 **	0.987	0.722
TPF 9	1100.75	1013.31	=0.001 *	0.929	0.588

Note: * p < 0.05, ** p < 0.01.

**Table 4:** ANOVA table for body mass index (BMI) and tests of physical fitness (TPF) grouped by weight percentile (underweight, normal weight value, overweight or obese).

	Mean	SD	F	*p*-Value
BMI	20.049	2.350	95.482	<0.001 **
TPF 1	1.19	0.176	1.927	0.128
TPF 2	3.414	0.662	2.157	0.096
TPF 3	2.999	0.751	4.000	0.009 *
TPF 4	14.912	3.597	1.208	0.309
TPF 5	3.951	0.622	2.125	0.100
TPF 6	12.999	0.751	4.000	0.009 *
TPF 7	21.419	2.433	2.863	0.039 *
TPF 8	4.559	0.512	0.683	0.564
TPF 9	1055.760	153.639	3.920	0.010 *

Note: * p < 0.05, ** p < 0.01. SD: Standard deviation. TPFs: (1) standing broad jump; (2) two leg jump (7 m); (3) single foot hop (7 m); (4) tennis ball throw; (5) 1 kg ball push; (6) wall bar climb; (7) 10 × 5 relay run; (8) 20 m sprint; and (9) 6 min lap run (reduced Cooper test).

**Table 5:** Effect size of weight percentile (underweight, normal weight value (NWV), overweight or obese) for variables with significant differences: body mass index (BMI) and tests of physical fitness (TPF) 3 (one foot hop, 7 m), 6 (1 kg ball push), 7 (10 × 5 relay run) and 9 (6 min lap run).

	Mean (SD)						
	Underweight	NVW	Overweight	Obese	*p*	1-β	*f*
BMI	14,428 (0.425) ^a^	18,947 (1,086) ^b^	21,613 (0.881) ^c^	24,545 (3,351) ^d^	<0.001 **	1	0.822
TPF 3	4,320 (3,224) ^a^	2,948 (0.706) ^b^	2,934 (0.423) ^b^	3.260 (0.393)	0.009 *	0.954	0.286
TPF 6	12,320 (3,224) ^a^	12,948 (0.706) ^b^	12,934 (0.423) ^b^	13.260 (0.393)	0.009 *	0.954	0.286
TPF 7	24,066 (3,550) ^a^	21,584 (2,315) ^a^	20,680 (2,521) ^b^	20,040 (2,129) ^b^	0.039 *	0.865	0.244
TPF 9	1105.000(153.052)	1082,294(161,745)^a^	1028.717(99.450)	933.181 (187.806) ^b^	0.010 *	0.950	0.283

Note: * p < 0.05, ** p < 0.01. SD: Standard deviation. ^a–d^ Different letters indicate statistically significant differences.

**Table 6:** Bivariate correlations between variables indicating health level and self-perception of physical fitness and motivation towards the practice of physical activity.

			PACES		IFIS	TPF Trial								
		BMI	EPA	BPA		1	2	3	4	5	6	7	8	9
**BMI**	Coef. correlation	--												
	Sig. (bilateral)	.												
**EPA (PACES)**	Coef. correlation	−0.128	--											
	Sig. (bilateral)	0.135	.											
**BPA (PACES)**	Coef. correlation	0.058	−0.241 **	--										
	Sig. (bilateral)	0.499	0.004	.										
**IFIS**	Coef. correlation	−0.65	0.349 **	−0.169 *	--									
	Sig. (bilateral)	0.451	0.000	0.047	.									
**TPF 1.**	Coef. correlation	−0.019	0.148	0.354 **	0.095	--								
	Sig. (bilateral)	0.825	0.083	<0.001	0.267	.								
**TPF 2.**	Coef. correlation	−0.113	−0.145	−0.014	−0.140	−0.011	--							
	Sig. (bilateral)	0.188	0.089	0.870	0.102	0.895	.							
**TPF 3.**	Coef. correlation	0.051	−0.095	0.021	0.185 *	−0.143	0.491 **	--						
	Sig. (bilateral)	0.553	0.266	0.805	0.030	0.095	<0.001	.						
**TPF 4.**	Coef. correlation	−0.028	0.011	−0.066	0.052	0.511 **	−0.140	−0.119	--					
	Sig. (bilateral)	0.747	0.895	0.439	0.547	<0,001	0.103	0.163	.					
**TPF 5.**	Coef. correlation	0.139	0.021	0.146	0.099	−0.314 **	−0.270 **	−0.091	0.007	--				
	Sig. (bilateral)	0.105	0.809	0.087	0.248	<0.001	0.001	0.289	0.936	.				
**TPF 6.**	Coef. correlation	0.051	−0.095	0.021	0.185 *	−0.143	0.491 **	1.000 **	−0.119	−0.091	--			
	Sig. (bilateral)	0.553	0.266	0.805	0.030	0.095	<0.001	0.000	0,163	0.289	.			
**TPF 7.**	Coef. correlation	−0.079	0.138	0.087	0.058	−0.112	0.262 **	0.242 **	−0.313 **	−0.318**	0.242**	--		
	Sig. (bilateral)	0.359	0,107	0.308	0.500	0,191	0.002	0.004	<0,001	<0,001	0.004	.		
**TPF 8.**	Coef. correlation	0.206 *	0.196 *	0.126	−0.058	−0.373 **	0.197 *	0.076	−0.441 **	−0.066	0.076	0.367 **	--	
	Sig. (bilateral)	0.015	0.021	0.140	0.501	<0.001	0.020	0.378	<0,001	0.439	0.378	<0.001	.	
**TPF 9.**	Coef. correlation	−0.269 **	0.260 **	−0.160	0.164	0.306 **	−0.105	−0.187 *	0.386 **	0.052	−0.187 *	−0.321 **	−0.360 **	--
	Sig. (bilateral)	0.001	0.002	0.061	0.254	<0.001	0.222	0.028	<0.001	0.543	0.028	<0.001	<0.001	.

Note: * p < 0.05, ** p < 0.01. IFIS: International Fitness Scale - self-reported fitness in youth. PACES: Physical Activity Enjoyment Scale. EPA: Enjoyment of Physical Activity. BPA: Boredom with Physical Activity. BMI: Body Mass Index. Tests of Physical Fitness (TPF): (1) standing broad jump; (2) two leg jump (7 m); (3) single foot hop (7 m); (4) tennis ball throw; (5) 1 kg ball push; (6) wall bar climb; (7) 10 × 5 relay run; (8) 20 m sprint; and (9) 6 min lap run (reduced Cooper test).

## Data Availability

Not applicable.
